# Systemic RNAi Delivery to the Muscles of ROSA26 Mice Reduces *lac*Z Expression

**DOI:** 10.1371/journal.pone.0102053

**Published:** 2014-08-15

**Authors:** Jessica Wei, Joel R. Chamberlain

**Affiliations:** Division of Medical Genetics, Department of Medicine, University of Washington, Seattle, Washington, United States of America; University of Pittsburgh School of Medicine, United States of America

## Abstract

RNAi has potential for therapeutically downregulating the expression of dominantly inherited genes in a variety of human genetic disorders. Here we used the ROSA26 mouse, which constitutively expresses the bacterial *lac*Z gene in tissues body wide, as a model to test the ability to downregulate gene expression in striated muscles. Recombinant adeno-associated viral vectors (rAAVs) were generated that express short hairpin RNAs (shRNAs) able to target the *lac*Z mRNA. Systemic delivery of these rAAV6 vectors led to a decrease of β-galactosidase expression of 30–50-fold in the striated muscles of ROSA26 mice. However, high doses of vectors expressing 21 nucleotide shRNA sequences were associated with significant toxicity in both liver and cardiac muscle. This toxicity was reduced in cardiac muscle using lower vector doses. Furthermore, improved knockdown in the absence of toxicity was obtained by using a shorter (19 nucleotide) shRNA guide sequence. These results support the possibility of using rAAV vectors to deliver RNAi sequences systemically to treat dominantly inherited disorders of striated muscle.

## Introduction

RNAi is a post-transcriptional gene silencing phenomenon that is a form of antiviral immune response mounted by many higher eukaryotes such as plants, nematodes, and insects. However, its primary role in mammals appears to be the processing of microRNAs (miRNAs), regulatory small RNAs that are cleaved to form double-stranded small interfering RNAs (siRNAs) for direct inactivation (translational repression) or degradation of a cognate mRNA [1]. RNAi was originally found in plants and nematodes and is a conserved process in evolution [2]. Further studies showed that double-stranded RNA (dsRNA) was at least 10-fold more potent than sense or antisense RNA alone [3]. Processing of precursor molecules for generating dsRNA for RNAi begins with long miRNAs (pri-miRNAs, >1000 nucleotides) that are processed into shorter sequences of ∼60–120 nt (pre-miRNA) in the nucleus by Drosha [4], [5]. Synthetic short hairpin RNAs (shRNAs) enter the pathway in the nucleus upon their expression, bypassing Drosha, and are transported to the cytoplasm via exportin-5 where they are processed by an RNase III-like enzyme, Dicer [6], [7]. This cleavage produces a ∼21 nt siRNA that is double-stranded with 2 nt 3′ overhangs. One strand of this complex is loaded into the RNA-induced silencing complex, RISC, to guide RISC in homologous mRNA cleavage or translational repression through binding, but not cutting (dependent on the extent of complementarity) [8]. Recognition that synthetic siRNAs could efficiently and specifically inhibit gene expression in cultured cells created tremendous interest and rapid development of the RNAi technology for functional genetics and the use of RNAi to suppress undesirable gene expression for treatment of human disease [9]. The use of RNAi delivery technologies is now showing significant potential for treating a variety of human disorders [10]–[14].

For development of treatments dependent on long-term expression of RNAi shRNAs were modeled from naturally occurring miRNAs and could be expressed from RNA polymerase III (RNA pol III) promoters at high levels, since these promoters typically drive expression of non-translated functional RNAs such as tRNAs [15], [16]. An early study of long-term viral expression of shRNAs (19–25 nt recognition sequences) from recombinant vectors derived from adeno-associated virus (rAAV8) in mouse liver resulted in efficient and persistent suppression of target RNA [17]. An unfortunate toxicity was discovered relating to the majority, but not all, of the different constructs and many led to premature death. Toxicity was traced to competition for the protein exportin-5, responsible for nuclear export of endogenous pre-miRNA, and argonaut 2, a component of the RNA induced silencing complex (RISC), but was not related to inflammatory responses from the vector or from consequences of activation of innate defense pathways in response to dsRNA [18]–[20]. These findings suggested that RNAi can work as a therapeutic agent *in vivo*, but further studies are necessary to select effective shRNA sequences of appropriate length, adjust expression levels and to ensure tissue specificity for efficacy and safety [10], [21], [22].

To explore the use of RNAi delivery to muscle we tested delivery of shRNA sequences using AAV vectors. To date there have been few reports of the use of rAAV vectors for systemic RNAi delivery to mammals. Wallace et al. showed that vectors derived from recombinant AAV6 (rAAV6) could effectively downregulate genes following intramuscular injection into mouse models for FSHD [23], [24]. We previously showed that systemic delivery of rAAV6 could also be used to downregulate FRG1 transcripts body wide in the FRG1 overexpressing mouse model for FSHD [25]. rAAV6 is a serotype with high tropism for muscle *in vivo* that has shown great promise through its ability to transduce muscles bodywide in the mouse [26]–[28]. rAAV vectors persist *in vivo* almost exclusively as episomes and display long-term expression in non-dividing cells such as muscle [29], [30]. By coupling expression of shRNAs for RNAi with AAV6 body-wide muscle transduction we hypothesized that expressed proteins or RNAs associated with dominant disease could be reduced or eliminated to treat dominant genetic disease of muscle. We describe tests of this system using as a target the ROSA26 mouse, which constitutively expresses β-galactosidase body wide, including throughout the striated musculature [31].

## Materials and Methods

All animal studies were performed in accordance with policies approved by the University of Washington Institutional Animal Care and Use Committee under protocol 3333-01.

### Generation of shRNA vectors and injection of mice

A plasmid containing an effective shRNA against β-gal (21-mer recognition sequence; [32]) expressed from the mouse U6 promoter was cloned into the AAV plasmid pARAP4 [33]. pARAP4 expresses the marker gene alkaline phosphatase (AP) from the Rous Sarcoma Virus (RSV) promoter. We cloned the U6 β-gal shRNA upstream of the RSV-AP gene in pARAP4 to make pAAV/*lac*Z-shRNA21 (5′-AACGTGACCTATCCCATTACG-3′). A similar vector but with a 2 nucleotide truncation was also made in the same vector (pAAV/*lac*Z-shRNA19; 5′-AACGTGACCTATCCCATTA-3′). Recombinant AAV vectors were generated in human 293D cells using the packaging plasmid pDG6 and purified as described [26]. Purified vectors were systemically injected into ROSA26 mice (Jackson labs, Bar Harbor, ME) via the tail vein. Purified rAAV6/shRNAβ-gal constructs were delivered by IV injection of 1–2.8×10^12^ vector genomes (vg) into the tail vein of ROSA26 mice (n = 3 or 6 per time point).

### Small RNA Northern analysis

HEK293 cells were transfected with rAAVshRNA plasmids and total RNA was isolated using the mirVana miRNA Isolation kit (Life Technologies). Size separation of 25 µg of total RNA per sample was performed with electrophoresis on a Novex 15% TBE-Urea gels with 300 pg and 30 pg of antisense or guide equivalent oligonucleotide per RNAi plasmid loaded on the gel as positive controls. Gels were stained with ethidium bromide and photographed as a reference for equivalent loading and quality assessment of total RNA. RNA was electroblotted to 0.45 um Nytran SPC Membranes (Sigma-Aldrich). RNA-bound membranes were prehybridized with ULTRAhyb- Oligo Hybridization Buffer (Life Technologies). Ten pmoles of a 21-mer antisense oligonucleotide was labeled with 5 µCi/probe γ-^32^P ATP (New England Nuclear) by T4 polynucleotide kinase (New England Biolabs). The radiolabeled probe was added to hybridization buffer and the RNA bound membrane and incubated for 16 hours at 32°C. The membrane was washed for 15 minutes, followed by 3 washes for 5 minutes each in 2× SSC/0.5% SDS at 32°C and simultaneously exposed to a phosphor screen and BioMax XAR film (Kodak). Film and phosphor screens were exposed from approximately 7–10 days at room temperature before developing the film and scanning the phosphor screen on a Storm 860 Scanner (GE Healthcare).

### Analysis of mouse tissues

Mouse tissues were harvested and analyzed for morphology, gene expression and histology as described [26], [34], [35]. Cryosections from mouse tissue were stained and analyzed at 2, 4, 6, or 12 weeks post-injection and compared with age-matched at time 0 uninjected ROSA26 and/or C57BL/6 mice. All animal studies were performed in accordance with policies approved by the University of Washington Institutional Animal Care and Use Committee.

## Results and Discussion

**Figure 1:**
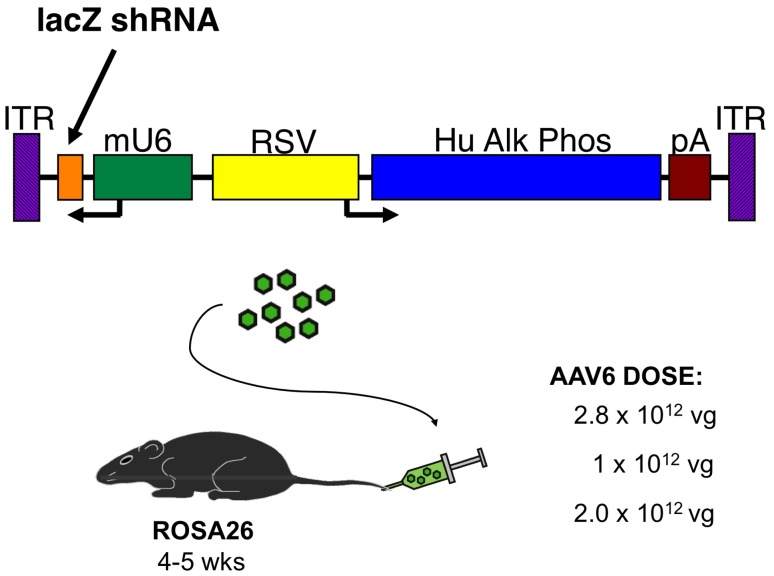
Schematic of an AAV genome carrying a *lac*Z shRNA expressed from the murine U6 promoter, together with an RSV-alkaline phosphatase cassette used to monitor vector transduction. No viral genes are present, and the inverted terminal repeats are the only portion derived from wildtype AAV. Single-stranded vector genomes are encapsidated following co-transfection of the vector genome plasmid and a helper plasmid into human 293D cells, and after purification are injected into the tail vein of mice.

To explore methods for treating dominant muscle disease *in vivo*, we used the ROSA26 mouse for testing systemic delivery of adeno-associated viral (AAV) vectors carrying RNAi expression cassettes. The ROSA26 mouse expresses *E. coli* β-galactosidase (β-gal) constitutively in the whole mouse from the endogenous ROSA26 promoter. Initially, a previously tested 21 nucleotide shRNA sequence directed against β-gal (kindly provided by Dr. Beverly Davidson [32]) and expressed from the mouse U6 promoter was introduced into a plasmid carrying the AAV2 inverted terminal repeats (ITRs) and packaging region. The murine U6 snRNA promoter (mU6) was chosen because it is a strong RNA polymerase III promoter and has been developed for expression of small RNAs, including expression of short hairpin RNAs for RNAi [15], [16]. The advantages of using the mU6 RNA polymerase III promoter include the typically high expression level of small, stable RNAs, compared to RNA polymerase II promoter transcripts (*e.g.* the endogenous ROSA26 promoter) for maximum RNAi activity, the minimal requirements of sequence near the start of transcription, and the precise generation of transcript ends. To monitor transduction of the vector in target tissues, the AAV backbone also carried a human placental alkaline phosphatase (hPlAP) reporter gene regulated by the RSV promoter [33]. This *lac*Z-shRNA/hPlAP vector is shown schematically in Figure 1. For striated muscle targeting we packaged the construct into recombinant AAV vectors pseudotyped with the AAV6 capsid, previously shown to enable efficient whole body striated muscle gene transfer into adult mice [27].

**Figure 2:**
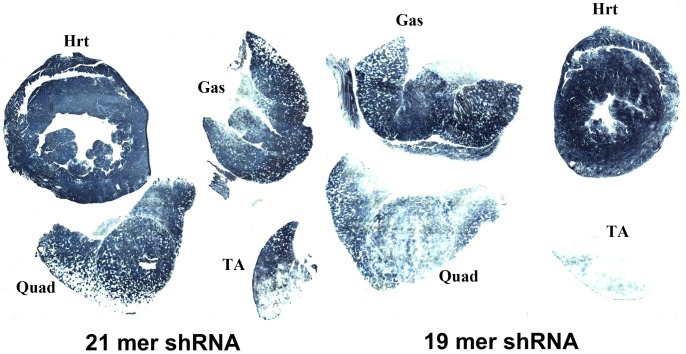
Systemic delivery of rAAV6 vectors expressing shRNAs can achieve body wide gene transfer to striated muscles. Heat-resistant human placental alkaline phosphatase staining of heart, *gastrocnemius*, *quadriceps* and *tibialis anterior* muscles of a mouse 6 weeks after tail vein injection of 2×10^12^ vector genomes of rAAV6/*lac*Z-shRNA21 (left) or rAAV6/*lac*Z-shRNA19 (right).

To test the ability to reduce β-gal expression in muscle we injected 5 week old ROSA26 mice with 1.0–2.8×10^12^ vector genomes (vg) of the rAAV6/*lac*Z-shRNA vector *via* the tail vein. Animals were sacrificed 2 and 4 weeks after the injection and monitored for transduction (by alkaline phosphatase [AP] expression), for β-gal expression and for histology. Histochemical staining of muscle cryosections for AP activity revealed widespread transduction of multiple striated muscle groups (Figure 2). Although rAAV6 vectors are able to target a number of muscle and non-muscle tissues (such as liver), significant levels of AP expression were not observed outside of striated muscle, as reported previously [27].

**Figure 3:**
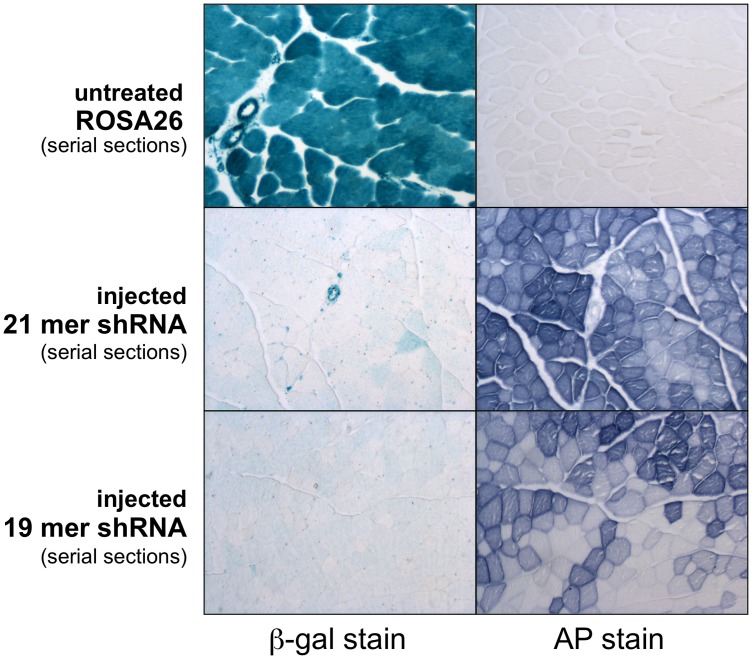
β-galactosidase and alkaline phosphatase expression in ROSA26 muscles. Shown are serial cryosections prepared from quadriceps muscles of untreated mice (top) or mice sacrificed 6 weeks after injection with 2×10^12^ vector genomes of rAAV6/*lac*Z-shRNA21 (middle) or rAAV6/*lac*Z-shRNA19 (bottom). Untreated muscles express high levels of β-gal (left), but do not express the heat-resistant human placental alkaline phosphatase enzyme (right). Both shRNA vectors effectively knock-down β-gal expression in myofibers expressing a variety of levels of AP, used as a marker for tissue transduction. Visible in the middle panel is a β-gal expressing artery and a number of capillaries, which are not transduced by AAV6.

A comparison of β-gal and alkaline phosphatase expression was used to assess the ability of the vector to knockdown β-gal expression in transduced muscles. β-gal expression was significantly reduced in quadriceps expressing the rAAV6 vector, as determined by a qualitative histochemical assay (Figure 3). Although a range of AP expression levels were observed in individual myofibers, β-gal expression was dramatically reduced in both high and low AP expressing cells (Figure 3).

**Figure 4:**
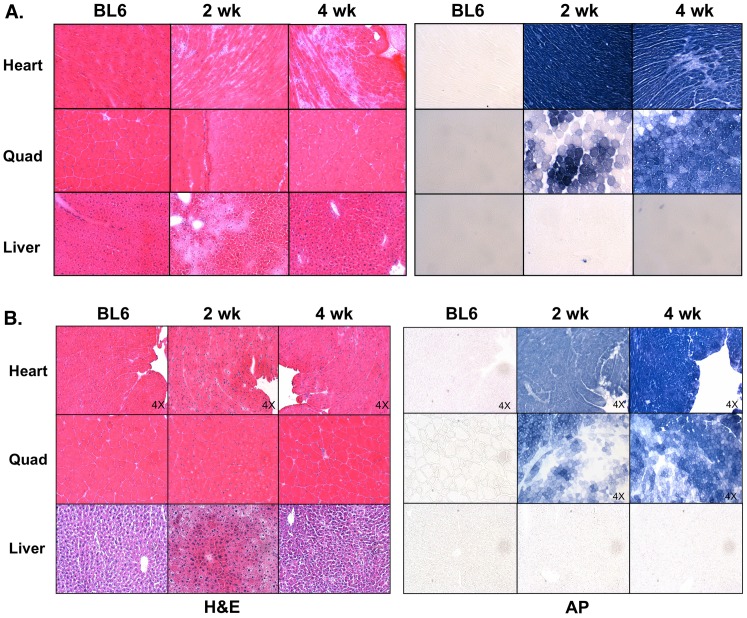
Histochemical analysis of cryosections from heart, quadriceps or liver of ROSA26 mice. Either untreated mice or mice injected with 2.8×10^12^ vector genomes (A) or 1×10^12^ vector genomes (B) of rAAV6/*lac*Z-shRNA21 were analyzed. On the left of each panel are shown hematoxylin and eosin stained sections, while on the right are shown sectioned stained for human placental alkaline phosphatase following heat-inactivation of the endogenous AP activity.

**Figure 5:**
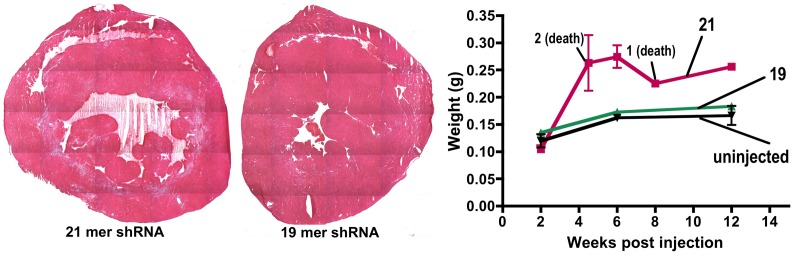
Analysis of cardiac muscle following systemic rAAV6/*lac*Z-shRNA delivery. A) Hematoxylin and eosin stained hearts from injection of the 21 and 19 nucleotide shRNA vectors showing a dilated cardiomyopathy that was mitigated by the shortened shRNA. Hearts were isolated 6 weeks after tail vein injection with 2×10^12^ vector genomes of rAAV6/*lac*Z-shRNA21 (left) or rAAV6/*lac*Z-shRNA19 (right). B) Cardiac weight in control and injected ROSA26 mice, obtained 2, 6 and 12 weeks post-injection. Note that several of the mice injected with the 21 nucleotide shRNA vector died, as indicated. All of the other mice persisted during the duration of the study.

Histological examination of quadriceps muscle from ROSA26 mice injected with the higher dose of vector (2.8×10^12^ vg) showed some indication of necrosis and regeneration in about 15% of skeletal myofibers, but significant areas of the heart and liver displayed necrotic regions (Figure 4A). Necrosis in the heart was most prominent proximal to the chambers at 2 weeks post injection and was more pronounced by 4 weeks (Figures 4A, 5A). By 5–6 weeks some injected mice began to die and a dilated cardiomyopathy was observed [Figures 5 (A, B)].

These results suggested that oversaturation of the natural miRNA pathway in the heart with this U6/β-gal shRNA expression cassette caused dilated cardiomyopathy similar to reports of knockout mice for Dicer and the Drosha complex protein DGCR8, the main processing enzymes of dsRNA for RNAi in the cytoplasm and nucleus, respectively [36], [37]. Toxicity from shRNA overexpression in the liver has previously been observed using a rAAV8 delivery system, which was attributed to oversaturation of exportin-5 and argonaut 2 in the natural RNAi pathway [17], [19]. Indeed, histochemical staining suggested that β-gal shRNA expression also caused transient liver damage in the ROSA26 mice that was resolved by 4 weeks (Figure 4A). These results do not appear attributable to either the AAV6 vector nor AP expression for 2 reasons: 1) little to no AP expression was observed in the liver (Figure 4), and 2) previous studies using systemic delivery of AP and a variety of other genes to striated muscles has not resulted in any observed liver or muscle toxicity, even at time points out to nearly 2 years [38]–[40].

**Figure 6:**
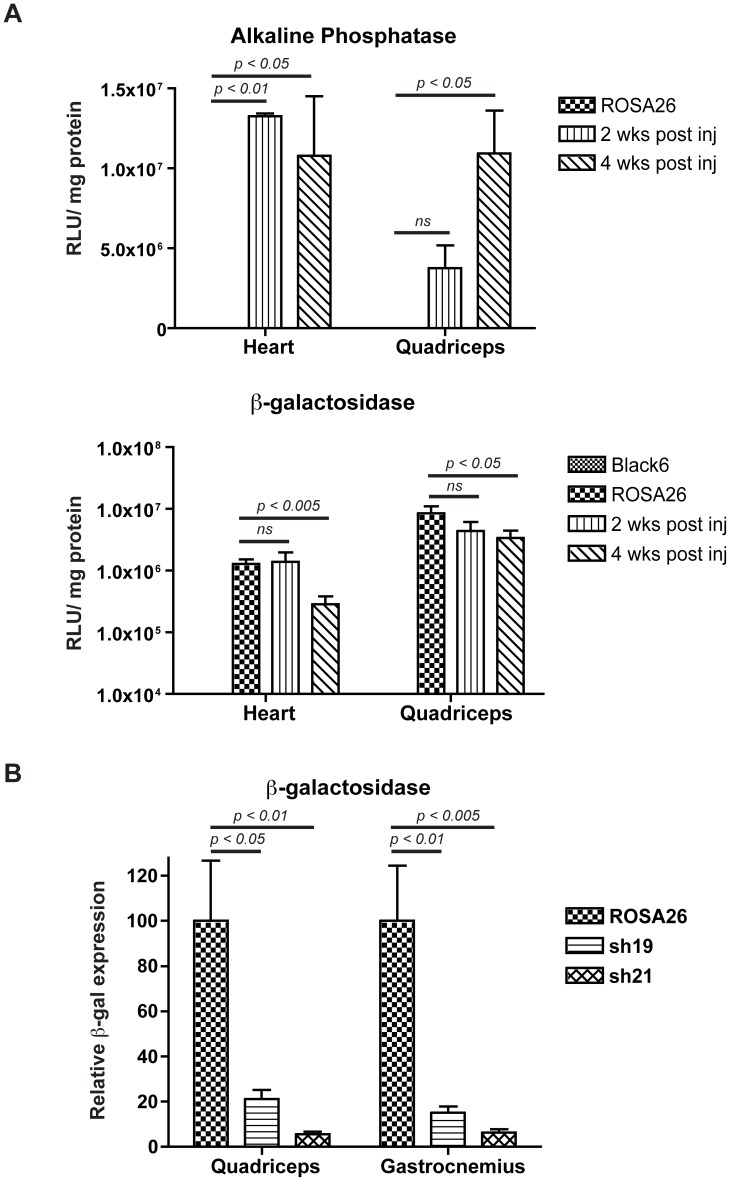
Systemic RNAi for β-gal knockdown. Enzymatic assays for quantitation of RNAi knockdown of β-gal and to detect viral transduction (AP). A) β-gal and AP activity in ROSA26 mouse heart and quadriceps muscles following systemic injection of 1×10^12^ vg of AAV6 β-gal shRNA (21 nucleotide target sequence). Tail vein injection was performed into ROSA26 mice at 5 weeks of age (n = 3 per time point), and analyzed 2 and 4 weeks post injection and compared with age-matched ROSA26 (positive control) and C57BL/6 mice (negative control). Note the log scale. B) Relative β-gal activity in ROSA26 mouse gastrocnemius and quadriceps muscles following systemic injection of 2×10^12^ vg of AAV6 β-gal shRNA (19 nucleotide target sequence). Analysis was at 6 weeks post-injection. Statistical analyses were performed using the Student's t test or 1-way ANOVA with Dunnett's multiple comparison test.

In contrast to the high dose injections, ROSA26 mice injected with a lower dose of vector (1×10^12^ vg) displayed vastly reduced regions of striated muscle necrosis (Figure 4B). However, a transient but marked necrosis was again observed in the liver at 2 weeks that had resolved by 4 weeks post-injection (Figure 4B). Lower vector doses were able to affect a significant reduction in β-gal activity in quadriceps and heart muscles (Figures 3, 6A). Importantly, little myonecrosis was observed either in quadriceps or the heart (Figures 4B, 5).

Tissue homogenates from muscle isolated at 2 and 4 weeks post-injection were prepared to quantitate the degree by which β-gal expression was reduced by the shRNA vector. Using a dose of 1.0×10^12^ vg both quadriceps and cardiac muscles displayed a marked reduction of β-gal expression 2 weeks post-injection, which reached levels 30-fold lower (quadriceps) and 50-fold lower (heart) than in non-transduced muscles by 4 weeks (Figure 6A). While AP activity dropped by ∼20% between the 2 week and 4 week time points in heart, the β-gal activity dropped >95%, indicating that any residual muscle necrosis was not enough to account in the decrease in β-gal activity (Figures 4B, 6A). Thus, while there is toxicity associated with expression of the β-gal shRNA, knockdown of β-gal activity is efficient with this vector. These results are the first to suggest that shRNA expression is able to interfere with the functioning of natural miRNAs in the heart as well as in liver.

**Figure 7:**
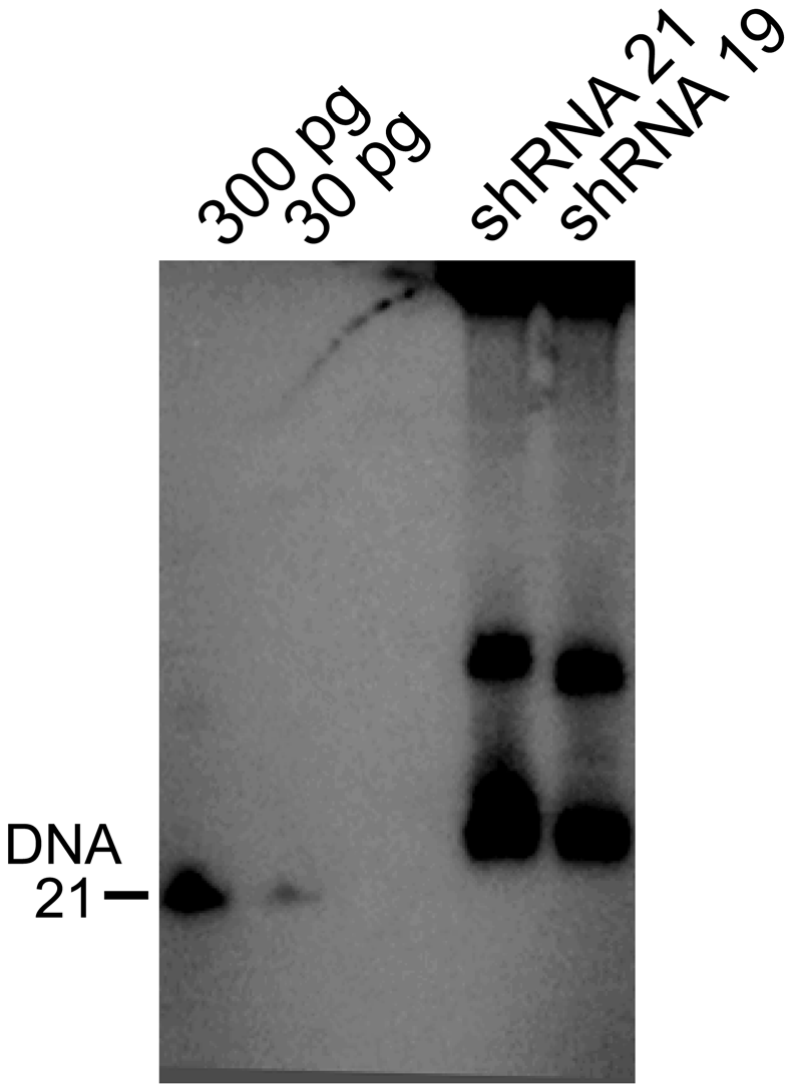
Comparison of siRNA levels produced from shRNA expression cassettes from rAAVshRNA plasmid transfected HEK293 cells. Small Northern analysis of 25 ug of total RNA size separated on a 15% denaturing acrylamide gel identified small precursor hairpins and siRNAs using radiolabeled DNA sense oligonucleotides. The first 2 lanes contain antisense DNA sequence identical to the 21-mer expression cassette corresponding to the shRNA guide strand included for siRNA size estimation.

We next asked whether altered delivery methods or vectors could be used to obtain efficient β-gal downregulation without myonecrosis. Previous studies showing liver toxicity with a variety of vectors carrying shRNA sequences 21–25 nucleotides also showed that shorter shRNA sequences often displayed no toxicity [17], [22]. We therefore developed a 19-mer shRNA sequence against β-gal and delivered it to ROSA26 mice using tail vein injection of 2×10^12^ vg of rAAV6 (Figure 1). Efficient transduction was again observed in a variety of striated muscles analyzed at 6 weeks, albeit at a somewhat lower level than with the higher vector dose (Figure 2). Following delivery of the longer 21 nucleotide vector β-gal activities were reduced by >90% in both quadriceps and gastrocnemius muscles by 6 weeks (n = 6), while the shorter 19-mer vector led to reductions of 79% in quadriceps and 85% in gastrocnemius muscles (n = 6; Figures 3, 6B and data not shown). While the 19 nucleotide vector enabled a significant reduction in β-gal activity in both heart and quadriceps (Figures 3, 6B), it did not lead to a dilated cardiomyopathy (Figure 5). A comparison of heart weights at necropsy for mice injected with either the 21 nucleotide or the 19 nucleotide shRNA vectors dramatically illustrates the dilated cardiomyopathy resulting from delivery of the 21 nucleotide vector, which also led to death of several of the injected mice 4–8 weeks post-injection. Importantly, none of the mice injected with the 19 nucleotide vector developed cardiomyopathy or died during the duration of the study (Figure 5). Furthermore, analysis of potential liver toxicity in the mice injected with the lower dose of vector revealed no elevation of either serum alanine transaminase (ALT) or aspartate aminotransferase (AST) levels in serum obtained 2, 6 or 12 weeks post-injection (data not shown). The 19 nucleotide vector also resulted in a somewhat lower level of knockdown than the 21 nucleotide vector, suggesting that the 19-mer may not be expressed as well. To test this point we transfected the 19 and 21 nucleotide vectors into HEK293 cells and directly measured shRNA levels by northern blot. As shown in Figure 7, the 19-mer siRNA (lower band) was expressed at a level 51% of the 21-mer siRNA (lower band).

The ability to knockdown expression of β-gal in a variety of muscles in the ROSA mouse is encouraging for future prospects for systemic RNAi delivery with AAV6. Several additional improvements for systemic RNAi using rAAV vectors can be envisioned. For example, the choice of the specific sequence encoded by the shRNA can affect not only siRNA efficiency but can also modulate overall toxicity [10], [17], [22]. In this regard we have recently shown that two separate shRNA sequences are effective at targeting the FRG1 transgene in a mouse model for FSHD [25]. Recent results in several systems suggest that miR30-based hairpins may display less toxicity than vectors based on simpler shRNA hairpins [41], [42]; JRC et al, submitted. Also, skeletal muscle specific expression of RNAi expression cassettes will be critical for lowering expression in the heart [43], [44]. Finally, dozens of different serotypes of AAV have ben described that display different tropisms for muscle and non-muscle tissues [45], [46]. While rAAV vectors based on serotypes 6, 8 and 9 have shown considerable promise for muscle delivery, all show various targeting of liver and several non-muscle tissues [47]. However, methods to generate hybrid or synthetic capsids proteins have the potential for either liver de-targeting, or enhancing delivery more specifically to muscle tissues [48].

## Conclusions

Systemic delivery of shRNA using AAV6 is an effective approach to RNA knockdown in muscle *in vivo* and led to a 50-fold reduction of β-gal activity in cardiac muscle and a 30-fold reduction in skeletal muscle in a transgenic mouse that constitutively expresses β-gal (ROSA26). Injection of rAAV/*lac*Z-shRNA21 resulted in cytotoxic effects in the liver and heart that appear to contribute to morbidity in some mice and is consistent with studies indicating oversaturation of the microRNA pathway [17]. Shortening the shRNA target recognition sequence resulted in an improved vector in regards to toxicity in both the liver and heart. Together these results support the possibility of using rAAV vectors for treating dominantly inherited muscle disorders.
